# MicroRNA-21 Overexpression Promotes the Neuroprotective Efficacy of Mesenchymal Stem Cells for Treatment of Intracerebral Hemorrhage

**DOI:** 10.3389/fneur.2018.00931

**Published:** 2018-11-06

**Authors:** Heyu Zhang, Yanzhe Wang, Qing Lv, Jun Gao, Liuting Hu, Zhiyi He

**Affiliations:** Department of Neurology, The First Affiliated Hospital of China Medical University, Liaoning, China

**Keywords:** mesenchymal stem cells (MSCs), microRNA-21, intracerebral hemorrhage, exosomes, TRPM7

## Abstract

Intracerebral hemorrhage (ICH) has high morbidity and mortality, with no effective treatment at present. One possible therapeutic strategy involves the use of mesenchymal stem cells (MSCs), which have shown promise in experimental models and have great potential for treating nervous illnesses in humans. However, many deficiencies in MSC treatment still need to be addressed, including their poor survival rate post-transplantation. Previously, we reported that the microRNA-21 (miR-21) is downregulated in ICH patients' blood and brain tissue. In this study, we aimed to examine its role and therapeutic efficacy in ICH using miR-21-overexpressing MSCs. We found that this microRNA can enhance MSC survival and recovery of neurological function in ICH rats. Its mechanism of action involves reduced neuronal apoptosis. In addition, we demonstrated that miR-21 can be transported to neurons through exosomes derived from MSCs and that it can target transient receptor potential melastatin 7 (*TRPM7)* to alleviate neuronal injury following ICH. We also observed that the NF-κB pathway is involved in the regulation of miR-21 in neural cells. In conclusion, miR-21 significantly enhances the survival of MSCs in ICH, and miR-21-overexpressing MSCs clearly improved neurological function in ICH rats. Transplantation of miR-21-overexpressing MSCs may, therefore, provide an effective strategy for neuroprotection and treatment of cerebrovascular diseases.

## Introduction

Arising from cerebrovascular rupture, ICH is one of the leading causes of morbidity and mortality worldwide (1). In the process of ICH, the initial injury is formed by the mechanical force of the expanding hematoma, and secondary injury usually involves neuronal death, damage to the blood-brain barrier, microglial activation, and astrocyte proliferation (2). Accumulating recent evidence has demonstrated that hemoglobin from hematoma, and its oxidative product hemin, play an essential role in autonomous and non-cell autonomous neuronal injury after the hemorrhagic event, exacerbating the inflammatory response and neuronal cell death (3). Because of its complex process, efficient treatments for ICH have been limited. However, in recent years, stem cell therapy has shown increasing promise as a strategy to aid recovery.

Mesenchymal stem cells (MSCs) are of great medical interest due to their multipotency, multilineage differentiation, and immune hyporesponsiveness (4, 5). Such unique properties make MSCs an ideal cell type for tissue regeneration and treatment of intractable diseases (6). For example, research has shown that MSC can migrate to a lesion and release a variety of cytokines that mediate angiogenesis and neuroprotective activities through paracrine signaling (7). Some remarkable pre-clinical studies have proved the therapeutic efficacy of MSC transplantation in neurological diseases (8, 9), especially acute injuries such as stroke (10, 11) and spinal injury (12). However, the microenvironment of ICH is not conducive for MSC survival or function. To enhance their therapeutic efficacy, interventions through endogenous genetic modification and exogenous treatment are necessary.

Studies have shown that miRNAs regulate neurological apoptosis, proliferation, differentiation, and autophagy (13, 14). Our previous study determined that miR-21 was significantly downregulated in peripheral blood and brain tissue of patients with ICH (15). Other studies have shown that miR-21 is upregulated in many tumor types, including glioma, hepatocellular carcinoma, breast and lung cancer, and plays an essential role in tumor cell apoptosis and autophagy by targeting tensin homolog deleted on chromosome ten (PTEN) (16–19). Moreover, Bhalala et al. demonstrated that miR-21 is upregulated in spinal cord injury (SCI), and alleviates the hypertrophic response to SCI in astrocytes (20). Accumulating evidence indicates that miR-21 also mediates the process of apoptosis, proliferation, and differentiation in multiple types of stem cells, which can enhance their therapeutic effect (21–24). Previous investigation has also shown that miR-21-overexpressing MSCs could help repair myocardial damage (25). However, the exact mechanism by which miR-21 acts after ICH remains to be elucidated.

In the present study, we aimed to investigate the effect of miR-21 on the functions of MSCs in treating ICH. We upregulated and downregulated miR-21 to demonstrate its role in hemin-induced MSC survival and the neuroprotective mechanism of miR-21 overexpression MSC in ICH rats. Our study demonstrates a novel therapeutic method to enhance the efficacy of MSCs in ICH.

## Materials and methods

### Animal preparation

All experimental protocols involving animals were performed according to the National Institutes of Health Guide for the Care and Use of Laboratory Animals and the ARRIVE (Animal Research: Reporting of *in vivo* Experiments) guidelines. Wistar rats (male, 250–280 g) used in this study were obtained from Liaoning Changsheng Biotechnology Co. Ltd (Liaoning, China). All rats were fed in a controlled environment (50% humidity, 22–25°C). Isoflurane was used for animal anesthesia. Efforts were made to minimize animal suffering and the number of animals used. Experimental procedures were conducted in accordance with the regulations of the animal protection laws of China and approved by the animal ethics committee of China Medical University (2012-38-1).

Rats were randomly divided into five groups, all of which underwent the same ICH surgical procedures: (1) Sham group (*n* = 42) rats underwent the same surgical procedures as rats in the control group without intracerebral injection; (2) Control group (*n* = 42) rats underwent the ICH surgical procedures and received vehicle intracerebral injection simultaneously when the treatment groups were administered MSCs of a different type; (3) MSC group (*n* = 42) rats received MSCs through intracerebral injection; (4) MSC-NC group (*n* = 42) rats were administered MSC-NCs through intracerebral injection; (5) MSC-miR-21 group (*n* = 42) rats received MSC-miR-21s via intracerebral injection. Next, 42 rats in each group were randomly divided into seven sub-groups by an investigator who was unaware of the neurological deficits of the rats. Six rats were decapitated on day 3, and 6 more on day 7 after ICH, to obtain fresh brain tissue samples to measure the water content. Six rats were perfused with fixative on day 3, and 6 more were perfused on day 7 after ICH, for histological preparation and analysis of the brain. Six rats were decapitated on day 3, and 6 more on day 7 after ICH, to obtain fresh brain tissue samples for biochemical analyses. Six rats were used for the neurological deficits scores until 14 days after ICH. All experimental data were collected and analyzed by an investigator who was unaware of the treatment administered to the rats.

### Rat model of intracerebral hemorrhage and assessment of neurological function

The ICH model was induced by stereotactic administration of 0.5 U bacterial collagenase type VII (Sigma Aldrich, USA) as described previously (26). Rats were divided into five groups, with one control (sham), and four ICH groups receiving an intracerebral injection of saline, normal MSCs, MSCs infected with empty lentivirus (NC-MSCs), or MSCs infected with miR-21-overexpressing lentivirus (miR-21-MSCs). A total of 10^6^ MSCs in 10 μl of saline were transplanted by intracerebral injection 3.0 mm right-lateral to the midline, 1.0 mm posterior to the bregma, 5.0 mm in depth below the skull. Neurological behavioral assessments were made on days 3, 7, and 14 after ICH, using the corner test and forelimb placement experiment as previously described (27). The wet weight of each brain was immediately obtained using an electronic balance, following which the brains were dried at 100°C for 24 h to obtain the dry weight. Water content was calculated as previously describe (28).

### Cell culture

Wistar rat bone marrow mesenchymal stem cells (BMMSCs), which had been primarily isolated, cultured, and passaged no more than twice, were purchased from Cyagen Biosciences Inc. (Santa Clara, CA, USA). These cells were cultured in DMEM-LG (Gibco, USA.) with 10% fetal bovine serum (Gibco, Australia.). We cultured PC12 cells in RPMI1640 with 10% horse serum and 5% fetal bovine serum (Gibco, Wellington, New Zealand). We cultured 293T cells in DMEM with 10% fetal bovine serum (Gibco, Sidney, Australia).

### ICH conditions of MSCs *in vitro*

To simulate the condition of ICH *in vitro*, MSCs were cultured with 100 μM Hemin (MedChemExpress, Shanghai, China). To determine the appropriate duration of Hemin treatment, cells were cultured with Hemin for 0, 12, 18, and 24 h. Cell viability was then analyzed using a Cell Counting Kit-8 (CCK-8; Dojindo, Japan).

### Synthetic RNA oligonucleotides and transfection

Mimics and inhibitors of miRNA-21, nonsense sequence for miRNA negative control (NC), negative control inhibitors, siRNA for TRPM7, and nonsense sequence for siRNA were all purchased from Genepharma (Shanghai, China). We transfected MSC and PC12 cells with Lipofectamine 2000 (Invitrogen, United States) according to the manufacturer's instructions. All *in vitro* transfections in our study were transient, and cells were not harvested for the subsequent assays until 48 h after RNA oligonucleotide transfection. Transfections *in vivo* were performed using lentivirus miR-21 and lentivirus NC, all supplied by Genepharma (Shanghai, China). The sequences of RNA oligonucleotides are listed in Supplement Table 1.

### RNA extraction and quantitative reverse transcription-PCR (qRT-PCR)

Total RNA was extracted from cells using TRIzol reagent according to the protocol (Invitrogen, United States). real-time PCR and miRNA RT-PCR were performed using a Hairpin-it miRNAs qPCR Quantitation Kit (GenePharma, Shanghai, China) according to the manufacturer's protocols. mRNA RT-PCR was performed using a PrimeScript RT Reagent Kit (TaKaRa Bio, Dalian, China). Real-time PCR was run on an ABI 7,500 using a QuantiTect SYBR Green PCR kit (TaKaRa Bio, Dalian, China). The relative miRNA expression of each gene was normalized to *U6* snRNA levels, and the relative mRNA expression of each gene was normalized to β*-actin* mRNA. The sequences of primers are listed in Supplement Table 2.

### Cell viability analyzed by CCK-8 assay

Cell viability was estimated by CCK-8 assay (Dojindo, Japan). MSCs were transfected with miR-21 mimics, miR-21 inhibitors, or negative control using Lipofectamine 2000 (Invitrogen, CA, United States) and cultured with Hemin for 18 h. We then added 10 μl CCK-8 reagent. Absorbance at 450 nm indicating cell viability was measured using an automated ELISA reader (SpectraMax® M5, Molecular Devices, United States).

### Flow cytometric analysis

After transfection with miR-21-5p mimics, inhibitors, and negative control, respectively, for 12 h, cell apoptosis was evaluated by quantitative detection of externalized phosphatidylserine using an Annexin V/FITC and PI apoptosis detection kit (BD Biosciences, San Jose, CA). Cells were incubated with hemin for 18 h, washed twice with cold PBS solution and suspended in 1x binding buffer. The cell suspension (100 μl) was then incubated with Annexin V-FITC (5 μl) and propidium iodide (PI; 5 μl) for 15 min at 25°C. This was followed by addition of binding buffer (400 μl) to the cell suspension, and the samples were then analyzed within 1 h using a flow cytometer (BD, FACSAria, CA, United States).

### Protein extraction and western blot analysis

We washed the MSC and PC12 cells three times, and then lysed them with RIPA (Beyotime, Beijing, China) with a cocktail (Roche, Germany). Protein concentration was determined with a BCA Protein Kit (Thermo, IL, United States). Cell lysates were separated using 4–12% sodium dodecyl sulfate polyacrylamide gel electrophoresis (SDS-PAGE), and proteins were transferred to polyvinylidene difluoride (PVDF) membranes. The membranes were incubated in 1% BSA solution with primary antibodies including anti-cleaved caspase-3, anti-matrix metalloproteinase 2 (MMP2), anti-matrix metalloproteinase 9 (MMP9), anti-tissue inhibitor of metalloproteinase-1 (TIMP-1)TIMP1, anti-transient receptor potential melastatin 7 (TRPM7), anti-p65, anti-phospho-IkB, and anti-beta actin (Abcam, CA, United States) at 4°C overnight and then incubated with the secondary antibody for 1 h at 22–25°C. The protein bands were detected using ECL (Electro-Chemi-Luminescence) substrate with the chemiluminescence method (LAS-3000, FUJIFILM, Japan).

### Dual luciferase assays

We seeded 293T cells in 24-well plates, cultured then overnight, and transfected them with the wildtype and mutant TRPM7 promoter-luciferase plasmids (0.1 μg per well of pGV272-wt-TRPM7 or pGV272-mt-TRPM7 plasmids) mediated by Lipofectamine 2000 (Invitrogen, CA, United States). Meanwhile, cells were cotransfected with either 0.4 μg miR-21 mimics or 0.4 μg miR-21 NC (Genepharma, Shanghai, China). Transfection efficiency was standardized by cotransfection with 0.02 μg Renilla luciferase reporter (Genechem, Shanghai, China). Both firefly and Renilla luciferase activities were quantified using a dual-luciferase assay system (Promega, E1910).

### Hematoxylin-eosin staining

Hematoxylin-Eosin staining was performed with a staining kit (Solarbio, Beijing, China). Rat brain slices were washed three times with PBS and then stained with hematoxylin stains for 90 s, hydrochloric acid ethanol differentiation solution for 60 s, and eosin stains for 60 s. Dehydration was performed as previously described (27).

### Tunel assays

We performed terminal deoxynucleotidyl transferase dUTP nick end labeling (TUNEL) assays with an *in-situ* cell death detection kit (Roche, Germany). Frozen sections of brain tissue were stained according to the manufacturer's instructions. Briefly, slides were washed with PBS three times, fixed with 4% paraformaldehyde for 30 min, incubated in 0.1% Triton X-100 for 2 min, and then in TUNEL reaction mixture for 60 min at 37°C.

### Exosome isolation

We transfected MSCs with miR-21 mimics, miR-21 inhibitors, or negative control, and cultured them in DMEM-LG with 10% exosome-free FBS for 48 h. Exosomes were isolated from culture supernatants using an ExoQucik-TC isolation kit (System Bioscience, United States); (29). Briefly, supernatants were centrifuged at 3,000 × g for 15 min to remove cells and cell debris, and they were then incubated with exosome isolation reagent at 4°C overnight. After centrifugation at 1,500 × g for 30 min, the supernatants were discarded, and the blank pipes were centrifuged at 1,500 × g for 5 min. We isolated miRNA from exosomes using a miRcute miRNA isolation kit (TIANGEN, China) according to the manufacturer's protocols. Exosome protein levels were determined using a BCA protein kit. Following this, the expression level of miR-21 was determined by real-time PCR, and the expression levels of CD9, CD63, and CD81 were analyzed by western blot as described above.

### Cell staining

PKH26 is a kind of dye commonly used for tracking stem cells (30). We stained MSCs using PKH26 according to the manufacturer's instructions. In brief, 2 × 10^7^ MSC cells were resuspended and incubated with 2 × 10^−6^ M PKH26 reagent for 2-5 min, washed three times with completed medium, and cultured in fresh medium for 12 h. The supernatant was then collected to isolate exosomes derived from MSCs. The positive cells were identified, counted, and analyzed in the sections with the ImageJ software and the survival MSCs ratio was calculated according to the following formula: positive cells/all cells per section.

### Co-culture of MSCs with PC12 cells

We co-cultured PC12 cells with MSCs transfected with miR-21 mimics, miR-21 inhibitors, or negative control. After 48 h of co-culture, the PC12 cells were collected for evaluation of apoptosis, RNA isolation, and real-time PCR analysis.

### Fluo-4 assays

We transfected PC12 cells with miR-21 mimics, miR-21 inhibitors, and negative control 24 h before treatment with Hemin for 18 h. Fluo-4 reagent was incubated with PC12 for 1 h at 37°C, washed with serum-free medium three times, and then detected using an automated ELISA reader (SpectraMax® M5, Molecular Devices, USA) with appropriate wavelength settings (excitation at 485 nm, emission at 520 nm).

### Statistical analysis

The data from each assay are expressed as mean ± standard deviation. Statistical significance between two groups was assessed using a two-tailed Student's *t*-test (α = 0.05). Analysis of variance (ANOVA), followed by a Bonferroni or SNK test was performed for multiple comparisons. We also performed a prior power analysis using the G^*^Power 3.1.9.2 software with a significance level of 5% to ensure we used an adequate number of animals per group. This indicated a power level greater than 0.9. All data were analyzed using SPSS 23.0 software (IBM Corp., Armonk, NY, United States) and GraphPad Prism 5.0 (GraphPad Software, Inc., La Jolla, CA, United States). All the *in vitro* and *in vivo* assays were repeated at least three times. Results were considered statistically significant only when *p* < 0.05.

## Results

### miR-21 enhanced survival of hemin-induced MSCs

To investigate the role of miR-21 in apoptosis regulation in MSCs, we estimated apoptosis, cell viability in miR-21 mimics, inhibitors, or negative control-transfected MSCs with Hemin treatment.

**Figure 1:**
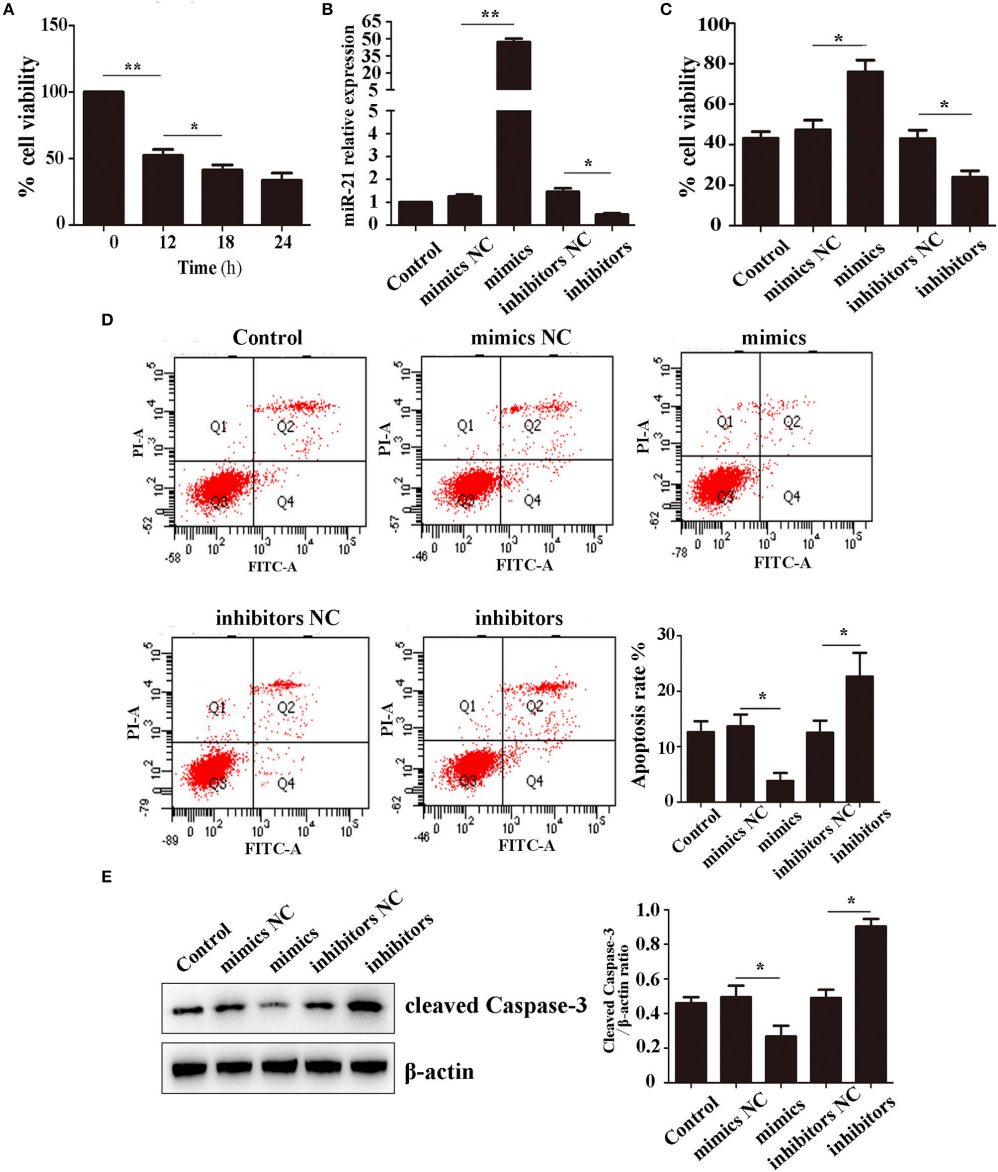
miR-21 enhanced survival of Hemin-induced mesenchymal stem cells (MSCs) **(A)**. Viability of MSCs treated with Hemin for different lengths of time **(B)**. Expression level of miR-21 detected by qPCR **(C)**. Viability of MSCs transfected with miR-21 mimics, miR-21 inhibitors, or their corresponding negative control (NC) **(D)**. Cell apoptosis analyzed by Annexin V-FITC and PI staining of MSCs in different groups **(E)**. Western blot analysis of cleaved-caspase-3 expression level in MSCs transfected with different miRNAs. ^*^p < 0.05, ^**^p < 0.01. Data are representative of three independent experiments.

We first detected the effect of Hemin on cell survival using CCK-8. After treatment with 100 μM Hemin, the rate of cell viability decreased in a time-dependent manner, reaching a minimum at 18 h (Figure 1A). Therefore, we decided to use 18 h treatments to further study the effects of miR-21 on Hemin-induced cellular apoptosis. We first transfected MSCs with miR-21 mimics, inhibitors, or negative control. The efficiency was evaluated with RT-PCR and western blot, the results of which demonstrated that transfection of miR-21 mimics elevated the expression of miR-21 more than 100-fold than that of the negative control, inhibitors of miR-21 reduced the expression by approximately 50%. The expression of PTEN, a known target gene of miR-21 was consistent with previous results (Figure 1B; Figure S4). We then showed that miR-21 enhanced the viability (Figure 1C) and decreased the apoptosis rate of MSCs with Hemin treatment, while miR-21 inhibitors showed the opposite effect (Figure 1D). Furthermore, we found that the level of cleaved-caspase-3, an important protein involved in the process of apoptosis, was downregulated in the miR-21 mimic group and upregulated in the miR-21 inhibitor group (Figure 1E).

### Enhanced neuroprotective effect of miR-21-MSCs in an ICH rat model

**Figure 2:**
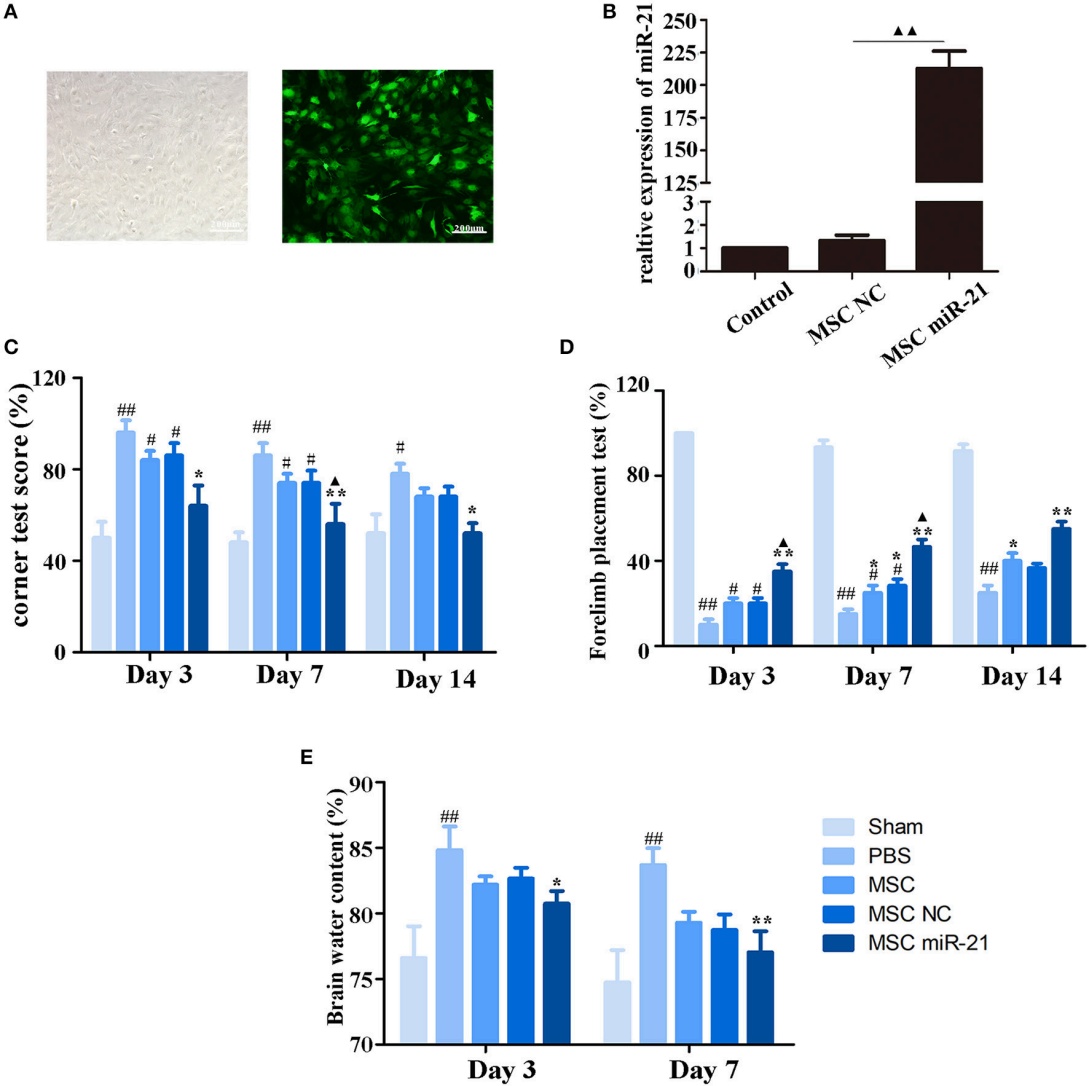
Enhanced neuroprotective effect of MSCs overexpressing miR-21 in ICH rats **(A)**. Morphology of MSCs transfected with miR-21 lentivirus (left panel, bright field; right panel, fluorescence field) **(B)**. Expression level of miR-21 detected by qPCR after lentivirus transfection **(C)**. Corner test scores on days 3, 7, 14 (*n* = 6) **(D)**. Forelimb placement test scores on days 3, 7, and 14 (*n* = 6; **E)**. Brain water content on days 3 and day 7 (*n* = 6). #*p* < 0.05 vs. sham; ^*^p < 0.05 vs. control; ^**^p < 0.01 vs. control; ▴*p* < 0.05 vs. MSC-NC; ^*##*^*p* < 0.01 vs. sham; ▴▴*p* < 0.01 vs. MSC-NC.

To investigate the therapeutic effect of miR-21-MSCs *in vivo*, MSCs were transfected with lentivirus-miR-21 or lentivirus-NC, and transfection efficiency was evaluated with real-time PCR (Figures 2A,B). The corner test and the forelimb placing test were performed on days 3, 7, and 14 after ICH to evaluate the neurological function of rats. The results showed that at days 3 and 7 after ICH, the scores of the corner test significantly decreased, and the scores of the forelimb placing test significantly increased in the PBS group compared with those in the sham group. However, both normal, negative control, and miR-21-overexpressing MSCs improved neurological function in ICH rats (Figures 2C,D). Furthermore, the neurological function of the miR-21 overexpressing group was improved compared with the NC group. In addition, the water content of the brain was also decreased in the miR-21-MSC group (Figure 2E).

### miR-21-MSCs effectively reduced hematoma area and cell apoptosis in ICH rats

**Figure 3:**
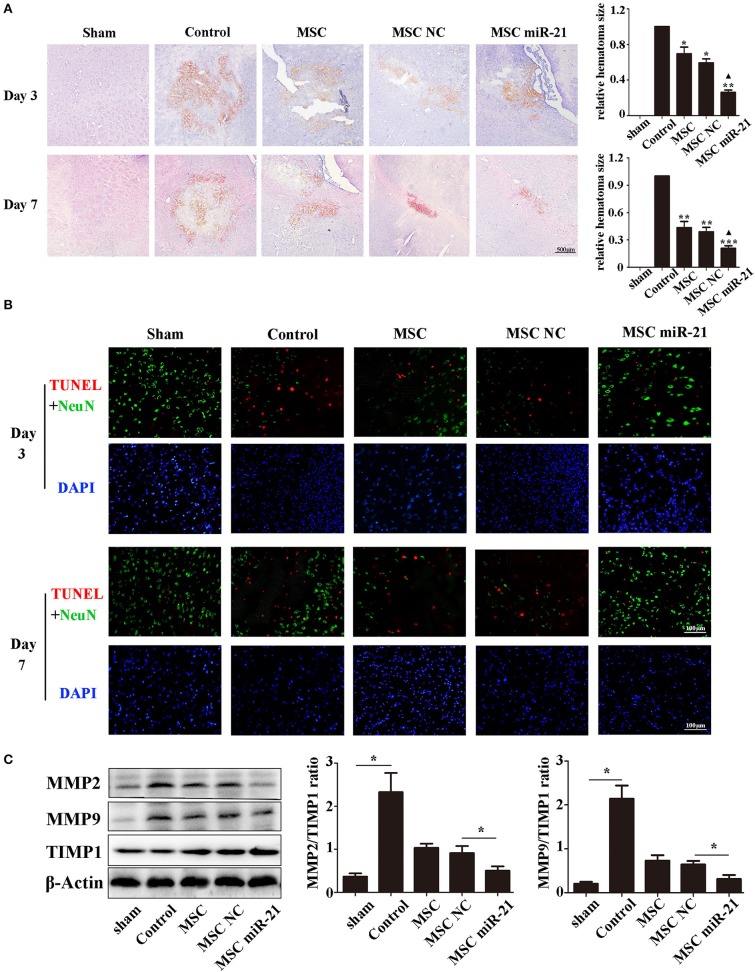
miR-21-overexpressing MSCs effectively reduce hematoma area and cell apoptosis in ICH rats **(A)**. Hematoxylin-eosin staining of hematoma **(B)**. Apoptotic cell death detected by terminal deoxynucleotidyl transferase dUTP nick end labeling (TUNEL) staining and fluorescence intensity (red) was decreased by MSC and miR-21 MSC **(C)**. Western blot analysis of the expression levels of MMP2, MMP9, and TIMP1 in MSCs transfected with different miRNAs. ^*^p < 0.05, ^**^p < 0.01. Data are representative of three independent experiments. ^***^p < 0.001; ▴*p* < 0.05 vs. MSC-NC.

To investigate the level of neuronal injury after ICH, we examined brain slices 3 and 7 days post-ICH. Histological results demonstrated shrinkage of the hematoma size in MSC-treated groups, especially the miR-21-MSC group (Figure 3A). TUNEL assays showed significantly reduced cell apoptosis in brain treated with miR-21-MSCs. These results demonstrated significantly reduced apoptosis of miR-21-MSCs compared to both PBS and NC-MSC groups, showing a better therapeutic effect of miR-21-MSCs on ICH (Figure 3B). Western blot analysis of MMP2, MMP9, and TIMP1 further confirmed the injury levels in the different groups described above (Figure 3C).

### miR-21 enhanced MSC viability in ICH rats

**Figure 4:**
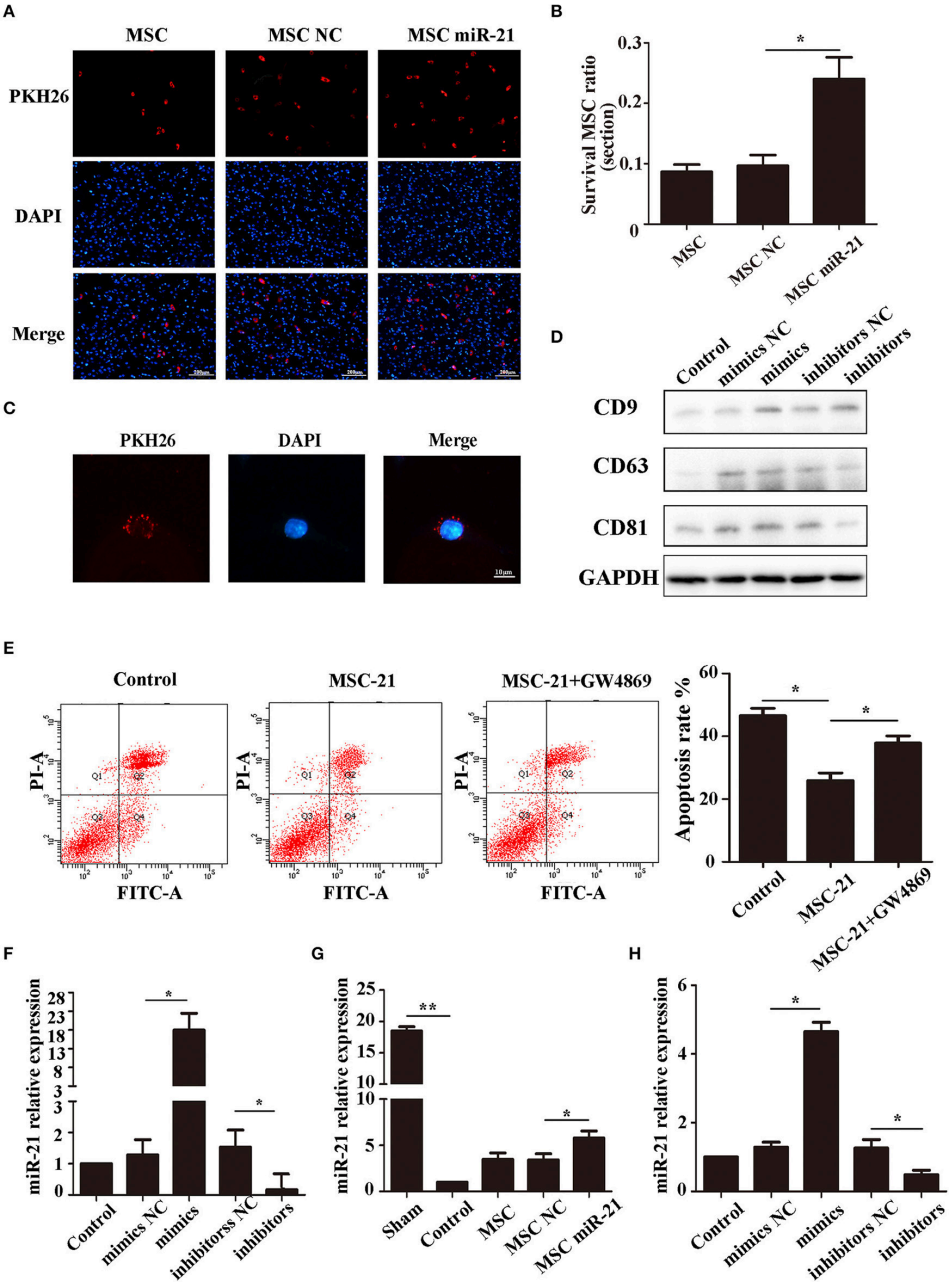
miR-21 enhanced viability of mesenchymal stem cells (MSCs) in ICH rats **(A)**. Surviving MSCs stained by PKH26 with red fluorescence **(B)**. The number of surviving MSCs in ICH rats **(C)**. MSC-derived exosomes can transfer to PC12 cells by membrane fusion **(D)**. Western blot-detected exosome markers CD9, CD63, and CD81 **(E)**. The anti-apoptotic effect of MSCs treated with GW4869 **(F)**. MiR-21 expression level in different groups' exosomes detected by qPCR **(G)**. MiR-21 expression level in co-cultured PC12 cells **(H)**. MiR-21 expression level in perihematomal tissue. ^*^p < 0.05, ^**^p < 0.01. Data are representative of three independent experiments.

The choke point of MSC therapy in ICH is that many MSCs die in the hemorrhagic environment. We, therefore, measured the survival of MSCs 3 days after injection. The cells in the brain were stained with DAPI, while the exogenous MSCs were stained with PKH26. Our results demonstrated that more miR-21-MSCs were retained compared with the other two groups (Figures 4A,B).

To investigate whether the role of miR-21 was mediated by exosomes, we isolated the exosomes of MSCs and co-cultured them with PC12 cells. The results showed that the exosomes could fuse with PC12 cells (Figure 4C), and the exosome markers CD63, CD81, and CD9 were used to verify exosomes (Figure 4D). Furthermore, the protective effect receded with the addition of GW4869, a common inhibitor of exosome secretion (Figure 4E). We measured the level of miR-21 in exosomes derived from miR-21-MSCs and NC-MSCs. Our results showed that miR-21 was significantly increased in miR-21-MSC-derived exosomes compared with the negative control (Figure 4F), and also significantly increased in co-cultured PC12 cells and the perihematomal tissue (Figures 4G,H).

### miR-21 could reduce PC12 cell death and TRPM7 was a function target of miR-21

**Figure 5:**
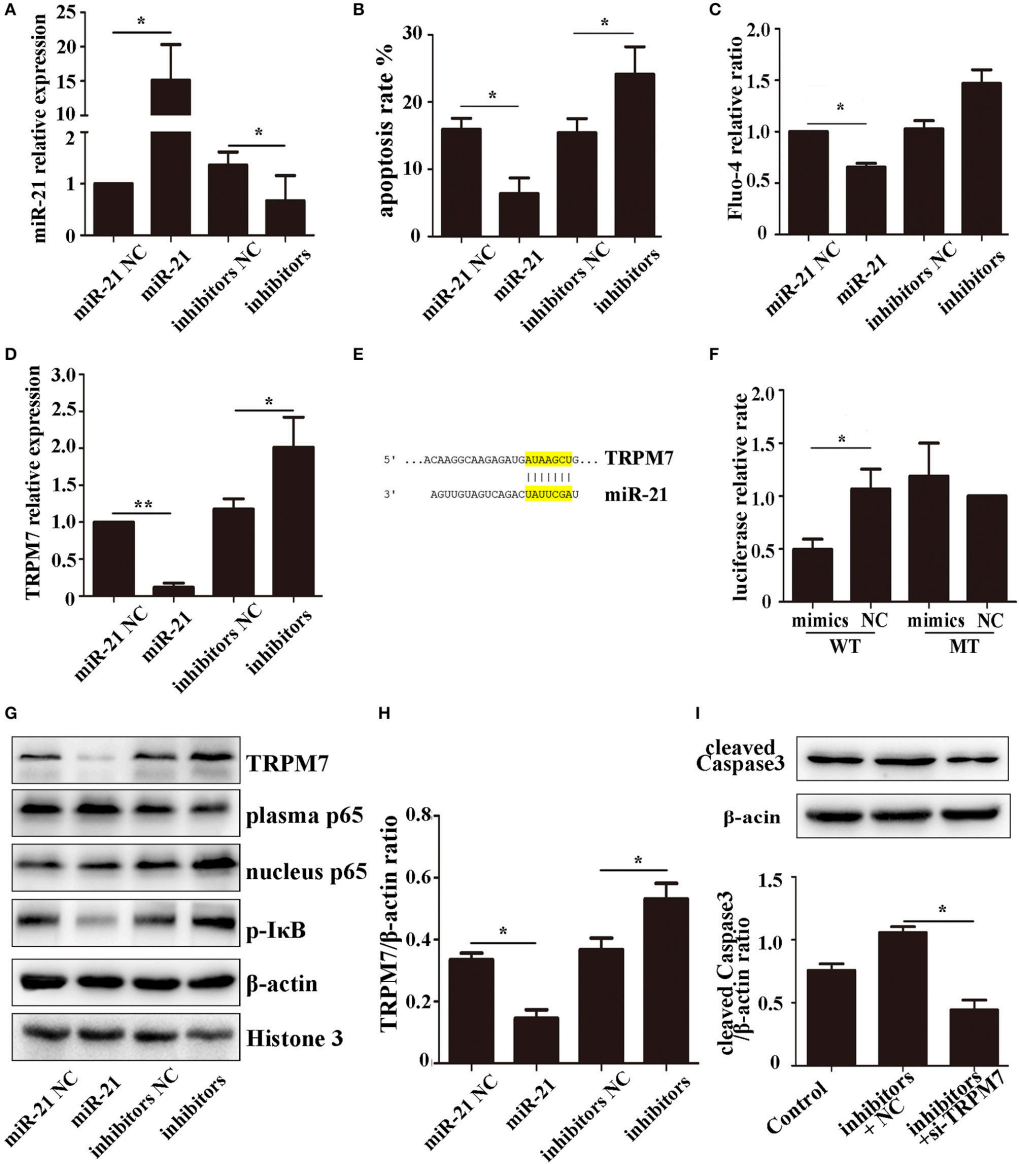
miR-21 can reduce PC12 cell death and *TRPM7* is a functional target of miR-21 **(A)**. MiR-21 transfection efficiency detected by qPCR **(B)**. Apoptosis rate of PC12 cells transfected with miR-21 mimics, miR-21 inhibitors, and their corresponding negative controls (NC) **(C)**. Fluo-4 assay of PC12 cells **(D)**. The expression level of *TRPM7* in PC12 cells transfected with miR-21 mimics, miR-21 inhibitors, and their corresponding negative controls (NC) **(E)**. Target region of miR-21 and *TRPM7* **(F)**. Luciferase assay for the combination of miR-21 and *TRPM7* **(G)**. Western blot analyzing the expression level of TRPM7, p65, and p-IκB proteins **(H)**. Quantitative histogram of TRPM7 expression level in different groups **(I)**. Western blot analyzing the expression level of cleaved caspase-3. ^*^p < 0.05, ^**^p < 0.01. Data are representative of three independent experiments.

To investigate the effect of miR-21 in PC12 cells, we determined the transfection efficiency (Figure 5A), apoptosis rate (Figure 5B, Figure S1), and Fluo-4 ratio (Figure 5C) of PC12 cells transfected with miR-21 mimics, miR-21 inhibitors, and negative control. The results showed that miR-21 can reduce PC12 cell death caused by Hemin treatment.

The expression of *TRPM7* mRNA was downregulated in PC12 cells transfected miR-21 (Figure 5D). We searched for potential direct mRNA targets of miR-21 using online databases and found a conserved binding site in the 3′-UTR region of *TRPM7* (Figure 5E, Figure S2). To investigate the relationship between miR-21 and *TRPM7*, we transfected miR-21 and pGV272-TRPM7 into 293T cells and detected the combination using dual-luciferase assays. The results showed that miR-21 could directly target and significantly down-regulate the expression of *TRPM7* (Figures 5F–H). Furthermore, we detected the expression of cleaved caspase-3 in PC12 cells transfected with miR-21 inhibitors and *TRPM7* siRNAs. The result showed that interference of *TRPM7* could partially reverse the effect of miR-21 inhibitors (Figure 5I), which revealed the role of *TRPM7* in the effect of miR-21.

### The NF-κB pathway is involved in the process of miR-21-mediated increased neuronal survival following hemin treatment

The NF-κB pathway plays a critical role in neuronal apoptosis in ICH (31). To determine if the role of miR-21 involves NF-κB pathway, we used western blotting to analyze the expression levels of the pathway components, p65 (plasma and nucleus) and p-IκB-α in PC12 cells. The results showed that miR-21 could decrease phosphorylation of IκB-α and decrease p65 transport to the nucleus (Figures 5G,H; Figure S3).

## Discussion

Intracerebral hemorrhage is characterized by primary hematoma formation and secondary injury, which gives rise to neural cell death and neurological dysfunction (32, 33). Apoptosis of neurons is one of the major features of the process of ICH. As a new tool for ICH therapy, MSCs have shown promise, but have tended to struggle or die in the ICH microenvironment (34). Previous research has also shown that miRNAs play an important role in the process of ICH (35, 36). In the present study, we demonstrated that miR-21 can protect MSCs from the toxicity of hemoglobin, while miR-21-overexpressing MSCs have an anti-apoptotic effect on neurons and improve neurological function in ICH rats.

In previous work, we found that the effect of miR-21 varies with different treatments and can act through multiple pathways (21, 37). Previous investigations of miR-21 have mostly been focused on its role in cancer; for example, it has been shown that miR-21 can affect the apoptosis and proliferation of glioblastoma cancer stem cells by targeting fas antigen ligand (*FASLG*) (21). It has also been shown to affect the resistance of hepatocellular carcinoma cells by inhibiting autophagy via the PTEN/Akt pathway (18). However, the role of miR-21 in MSCs is less clear. In this study, we explored the effect of miR-21 on MSCs in ICH. Here, we report that miR-21 significantly decreases MSC apoptosis with hemin treatment, which has been widely used to simulate the ICH microenvironment *in vitro* (38). Our results showed that miR-21 mimics can markedly decrease the rate of apoptosis and the expression of apoptosis-related proteins by MSCs in ICH. These data suggest that miR-21 could play a protective role in MSCs in the ICH microenvironment.

Other evidence that miRNAs can enhance the effectiveness of MSCs has recently been accumulating. Chen et al. reported that overexpression of miR-133 promotes the therapeutic efficacy of mesenchymal stem cells against acute myocardial infarction (39), and Shi et al. demonstrated that miRNA-486 regulates angiogenic activity and survival of mesenchymal stem cells under hypoxia (40). Furthermore, previous research has demonstrated that MSCs overexpressing miR-21 can help repair myocardial damage (25). We, therefore, hypothesized that miR-21 can enhance the neuroprotective effects of MSCs in ICH. Our results showed that miR-21 MSCs can enhance the recovery of neurological function compared with other groups, as evidenced by improved scores in the corner test and forelimb placement experiment. They could also shrink hematomas and reduce the number of neuronal apoptosis after ICH. Moreover, we found that miR-21-overexpressing MSCs could also increase survival after transplantation into ICH rats.

As previously demonstrated, the transplant and successful differentiation of MSCs is very rare (41, 42). The functions of MSCs are mainly achieved through paracrine signaling, including the release of cell factors and exosomes, which play parallel roles in intercellular communication (43, 44). The exosomes secreted from MSCs play various roles, such as accelerating skeletal muscle regeneration (45), inhibiting myocardial fibrosis (39), and increasing the chemosensitivity of hepatocellular carcinoma (46). In the present study, we found that exosomes secreted from MSCs could fuse with PC12 cells. The expression of miR-21 is significantly increased in exosomes secreted by miR-21-overexpressing MSCs compared with controls. In view of these results, we conclude that miR-21-overexpressing MSCs can prevent neuronal death from secondary injury after ICH, and that this is mediated by miR-21 derived from exosomes. Furthermore, we demonstrated that the expression of miR-21 remarkably increased in co-cultured PC12 cells and perihematomal tissue.

It is notable that downregulation of miR-21 has been demonstrated in brain tissue of ICH patients (15), and its overexpression was shown to act against neuronal apoptosis and protect the brain from trauma injury (37). However, the role of miR-21 in spontaneous intracerebral hemorrhage is unclear. We regulated the expression of miR-21 in PC12 cells, and the results revealed that miR-21 could significantly reduce hemin-induced neuronal death. An exciting result in relation to this was that *TRPM7*, a neuronal apoptosis gene, was predicted by miRWalk to be a target of miR-21. Our qPCR and dual luciferase results confirmed the regulatory effect of miR-21 on *TRPM7*. Many studies have shown that *TRPM7* not only has an ion-conduction property, but also kinase activity (47, 48). It has been demonstrated that *TRPM7* is directly linked to neuronal death, which involves activation of a non-selective cation channel with high permeability to Ca^2+^ (49, 50). Indeed, blocking TRPM7 channels or suppressing its expression by RNA interference was effective in preventing the death of neurons in stroke (51). Furthermore, previous studies have revealed that the NF-κB pathway may be involved in the process of neuronal death induced by ionic overload (52) and inhibiting its excitation may improve the prognosis of ICH (53). In line with these results, we found that miR-21 could reduce the expression of *TRPM7* with Hemin treatment and alleviate the activation of the NF-κB pathway, reducing the level of IκB-α phosphorylation and inhibiting p65 nuclear translocation. Furthermore, interference TRPM7 could reverse the effect of miR-21 inhibitors which partly confirmed that TRPM7 involved in process of miR-21.

However, there are a few limitations to the present study. The rat model of ICH induced by collagenase type VII could not completely simulate the pathological changes of ICH, and PC12 is a kind of pheochromocytoma derived cell line, which, despite not entirely replacing primary neurons, is commonly used as *in vitro* model of neuronal function and differentiation. In our study, we found that miR-21 could directly regulate the expression of *TRPM7* and the NF-κB pathway, which is involved in the neuroprotective effect mediated by exosome-derived miR-21. However, the reverse effect of interfering TRPM7 on miR-21 inhibitors was limited. Since there are so many potential target genes of miR-21, TRPM7 is a novel target and just taking part of the neuroprotective effect. In addition, the underlying mechanism by which *TRPM7* affected miR-21 neuroprotection and led to NF-κB-mediated signal transduction remains unclear. Further mechanistic studies investigating how *TRPM7* and NF-κB pathway are involved in miR-21-related neuroprotective process of ICH are required.

In conclusion, our present study demonstrated that miR-21 increased the survival of MSCs and enhanced the efficacy of MSC therapy in acute intracerebral hemorrhage. In addition, we observed that miR-21 could be transported from MSCs to neurons through exosomes and plays an important role in hemoglobin-induced neuronal death by targeting *TRPM7*. Furthermore, we demonstrated that miR-21 overexpression could alleviate the activation of the NF-κB pathway. Finally, our findings suggest that miR-21-overexpressing MSCs could provide a novel method for the treatment of ICH.

## Author contributions

YW: data curation and Formal analysis; HZ, QL, JG, and LH: investigation; ZH: funding acquisition, project administration. HZ: writing – original draft. All authors agree to be accountable for the content of the work.

### Conflict of interest statement

The authors declare that the research was conducted in the absence of any commercial or financial relationships that could be construed as a potential conflict of interest.
