# Supplementary material for: MicroRNA-21 Overexpression Promotes the Neuroprotective Efficacy of Mesenchymal Stem Cells for Treatment of Intracerebral Hemorrhage
Source: Front Neurol. 2018 Nov 6;9:931. doi: 10.3389/fneur.2018.00931 (PMC6233525; doi:10.3389/fneur.2018.00931)
Supplement: Supplementary file 1 [file Data_Sheet_1.docx]

Supplementary Material

MicroRNA-21 overexpression promotes the neuroprotective efficacy of mesenchymal stem cells for treatment of intracerebral hemorrhage

Zhang Heyu^1^, Wang Yanzhe^1^, Lv Qing^1^, Gao Jun^1^, Hu Liuting^1^, He Zhiyi^1,*^

*** Correspondence:** Prof. He Zhiyi: [hezhiyi0301@sina.com](mailto:hezhiyi0301@sina.com)

# Supplementary Data

Supplementary Material should be uploaded separately on submission. Please include any supplementary data, figures and/or tables. All supplementary files are deposited to FigShare for permanent storage and receive a DOI.

Supplementary material is not typeset so please ensure that all information is clearly presented, the appropriate caption is included in the file and not in the manuscript, and that the style conforms to the rest of the article.

# Supplementary Figures and Tables

For more information on Supplementary Material and for details on the different file types accepted, please see [here](http://home.frontiersin.org/about/author-guidelines#SupplementaryMaterial). Figures, tables, and images will be published under a Creative Commons CC-BY licence and permission must be obtained for use of copyrighted material from other sources (including re-published/adapted/modified/partial figures and images from the internet). It is the responsibility of the authors to acquire the licenses, to follow any citation instructions requested by third-party rights holders, and cover any supplementary charges.

## Supplementary Figures


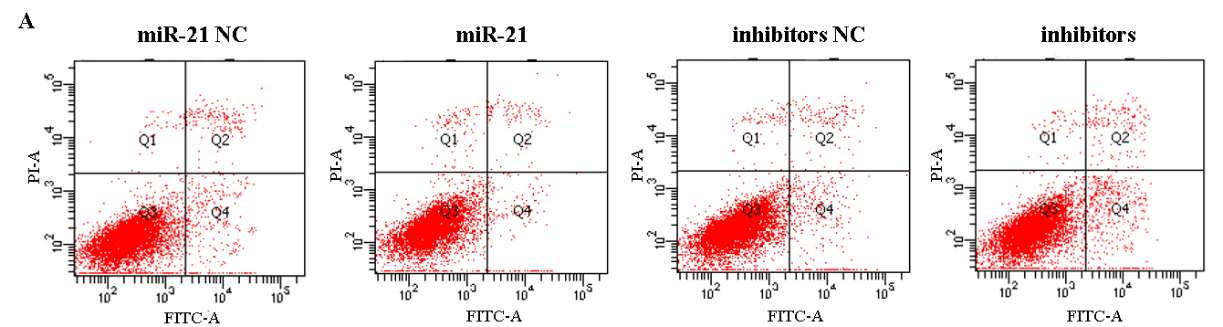


Supplementary Figure 1. Apoptosis rate of PC12 cells transfected with miR-21 mimics, miR-21 inhibitors and their corresponding negative control (NC).


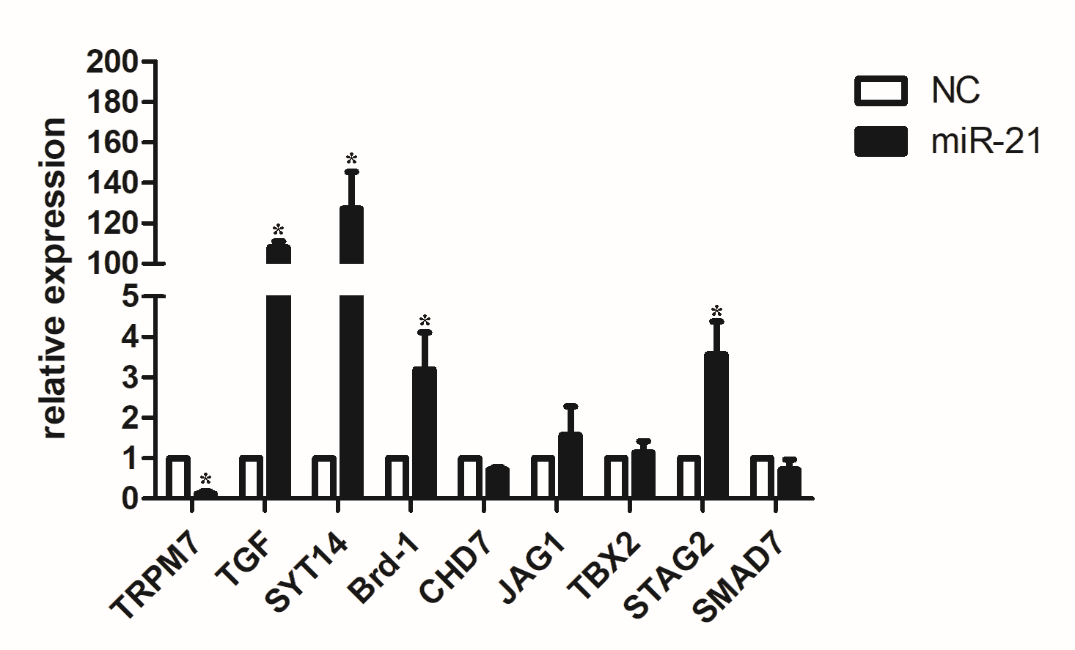


**Supplementary Figure 2.** Relative expression of potential target genes.


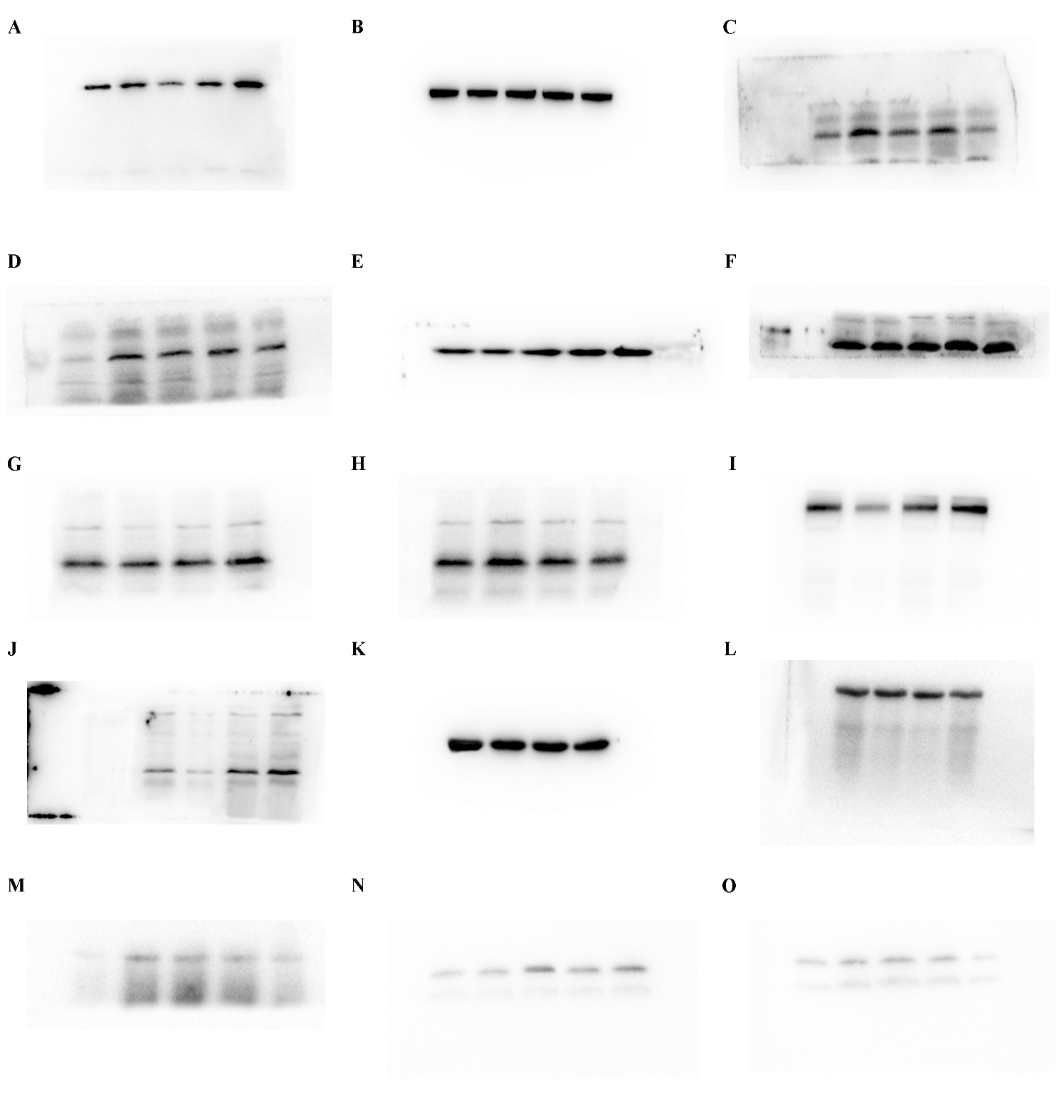


**Supplementary Figure 3**. Original Western Blotting. (A). Western blotting of cleaved-caspase 3. (B). Western blotting of β-actin. (C).Western blotting of MMP2. (D).Western blotting of MMP9. (E).Western blotting of TIMP1. (F).Western blotting of β-actin. (G). Western blotting of nucleus p65. (H). Western blotting of plasma p65. (I). Western blotting of p-IκB. (J).Western blotting of TRPM7. (K).Western blotting of β-actin. (L). Western blotting of Histone 3. (M). Western blotting of CD63. (N).Western blotting of CD9. (O). Western blotting of CD81.


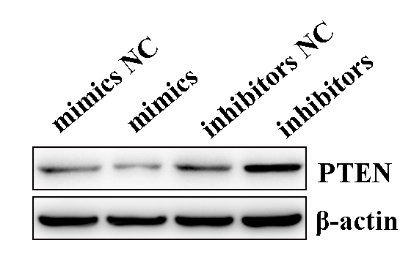


**Supplement Figure 4.** The expression of PTEN detecting by western blotting.

## Supplementary Tables

**Table 1. The sequence of miRNA oligoes**

|  | Sequence |
| --- | --- |
| miR-21 mimics | 5’-UAGCUUAUCAGACUGAUGUUGA-3’  5’-AACAUCAGUCUGAUAAGCUAUU-3’ |
| miR-21 inhibitors | 5’-UCAACAUCAGUCUGAUAAGCUA-3’ |
| miRNA negative control | 5’-UUCUCCGAACGUGUCACGUTT-3’  5’-ACGUGACACGUUCGGAGAATT-3’ |
| miRNA inhibitors negative control | 5’-CAGUACUUUUGUGUAGUACAA-3’ |
| siRNA TRPM7(-3820) | 5’-GGCUCAGAAUCUUAUUGAUUU-3’  5’-AUCAAUAAGAUUCUGAGCCUU-3’ |
| siRNA negative control | 5’-UUCUCCGAACGUGUCACGUTT-3’  5’-ACGUGACACGUUCGGAGAATT-3’ |

**Table 2. The sequence of primers**

|  | Forward | Reverse |
| --- | --- | --- |
| miR-21 | 5’-TCGCCCGTAGCTTATCAGACT-3’ | 5’-CAGAGCAGGGTCCGAGGTA-3’ |
| U6 | 5′-ATTGGAACGATACAGAGAAGATT-3′ | 5′-GGAACGCTTCACGAATTTG-3′ |
| TRPM7 | 5’-TGCCATCTGAAGGAGGAACA-3’ | 5’-ACTCTGCGACAGCCTCATCA-3’ |
| β-actin | 5′-TGTCACCAACTGGGACGATA-3′ | 5′-GGGGTGTTGAAGGTCTCAAA-3′ |
